# Identification of the Subtypes of Renal Ischemia-Reperfusion Injury Based on Pyroptosis-Related Genes

**DOI:** 10.3390/biom13020275

**Published:** 2023-02-01

**Authors:** Xinhao Niu, Yin Celeste Cheuk, Xiao Li, Ruiming Rong, Xiaoqing Xu, Cuidi Xu, Yongsheng Luo, Pingbao Zhang, Jingjing Guo

**Affiliations:** 1 Shanghai Key Laboratory of Organ Transplantation, Shanghai 200032, China; 2 Department of Urology, Zhongshan Hospital, Fudan University, Shanghai 200032, China; 3 Department of Urology, Huashan Hospital, Fudan University, Shanghai 200040, China; 4 Department of Cardiology, Zhongshan Hospital, Fudan University, Shanghai 200032, China; 5 Shanghai Institute of Cardiovascular Diseases, Shanghai 200032, China

**Keywords:** renal ischemia-reperfusion injury, renal transplant, pyroptosis, immune microenvironment, diagnostic model, non-negative matrix factorization

## Abstract

Ischemia-reperfusion injury (IRI) often occurs in the process of kidney transplantation, which significantly impacts the subsequent treatment and prognosis of patients. The prognosis of patients with different subtypes of IRI is quite different. Therefore, in this paper, the gene expression data of multiple IRI samples were downloaded from the GEO database, and a double Laplacian orthogonal non-negative matrix factorization (DL-ONMF) algorithm was proposed to classify them. In this algorithm, various regularization constraints are added based on the non-negative matrix factorization algorithm, and the prior information is fused into the algorithm from different perspectives. The connectivity information between different samples and features is added to the algorithm by Laplacian regularization constraints on samples and features. In addition, orthogonality constraints on the basis matrix and coefficient matrix obtained by the algorithm decomposition are added to reduce the influence of redundant samples and redundant features on the results. Based on the DL-ONMF algorithm for clustering, two PRGs-related IRI isoforms were obtained in this paper. The results of immunoassays showed that the immune microenvironment was different among PRGS-related IRI types. Based on the differentially expressed PRGs between subtypes, we used LASSO and SVM-RFE algorithms to construct a diagnostic model related to renal transplantation. ROC analysis showed that the diagnostic model could predict the outcome of renal transplant patients with high accuracy. In conclusion, this paper presents an algorithm, DL-ONMF, which can identify subtypes with different disease characteristics. Comprehensive bioinformatic analysis showed that pyroptosis might affect the outcome of kidney transplantation by participating in the immune response of IRI.

## 1. Introduction

In the process of kidney transplantation, ischemia-reperfusion injury (IRI) is an inevitable complication during kidney transplantation [1]. IRI may lead to the occurrence and development of subsequent acute kidney injury, thus affecting patients’ renal function and prognosis [2]. Therefore, it is essential to classify IRI, which will assist clinicians in evaluating the long-term prognosis of different patients.

To explore the role of ferritin-deficiency-related genes (FRG) in the classification of IRI, Wei et al. used consensus cluster analysis and the least absolute shrinkage and selection operator (LASSO) to construct predictive features of delayed graft function associated with FRG. They identified two iron-related patient clusters (pBECN1 and pNF2) in renal IRI samples [3]. Zhang et al. explore the mechanisms and potential molecular markers involved in renal IRI. They revealed the vital role of the NOD-like receptor signaling pathway during IRI and the close relationship between this pathway and the infiltration of specific immune cell types through a series of bioinformatics analyses [4]. Meng et al. identified immune-related genes associated with graft rejection and developed prognostic models based on immune-related genes in kidney transplantation. This model has good sensitivity and specificity in predicting 1-year and 3-year renal transplant survival [5].

Non-negative matrix factorization (NMF) is a classical clustering analysis method that obtains a low-dimensional representation of samples (features) by factoring the complete data matrix into the form of multiplication of two nonnegative matrices (basis and coefficient matrices). NMF algorithms are widely used in image recognition, electroencephalogram (EEG) signal processing, and biological data analysis. NMF algorithms make significant progress in disease classification in recent years [6,7]. Gong et al. explored the prognostic role of adjacent non-tumor tissues in patients with hepatocellular carcinoma (HCC) through the NMF algorithm. They found significant prognostic differences among different subgroups by analyzing the results of three subgroups [8]. Winterhoff et al. used Agilent microarrays to determine the gene expression profiles of 276 well-annotated ovarian cancers, four TCGA transcriptional isoforms, and their significant prognostic associations in all three histological subtypes (*p* < 0.001) [9]. Zhao et al. used the NMF approach to identify immunophenotypes and latent subtypes using 719 HCC samples with public genomic data. The authors divided hepatocellular carcinoma into high-risk and low-risk subtypes to provide clues for prognosis and immunotherapy prediction of hepatocellular carcinoma [10]. Alzheimer’s disease (AD) is a heterogeneous disease. Zheng et al. used the gene expression data of 222 AD patients in the Religious Order Study and the Memory and Aging Project (ROSMAP) study to identify two subtypes of AD, namely synaptic type and inflammatory type, using the NMF algorithm [11].

However, the traditional NMF algorithm does not consider the rich information in the data and the solution to cope with high-dimensional features in the clustering process. Therefore, this paper proposes an algorithm based on double Laplacian orthogonal nonnegative matrix factorization (DL-ONMF). In this algorithm, the interaction information of samples and features is considered, and the sample network and feature network are added to the algorithm by graph regularization. In addition, orthogonality constraints on the basis matrix and the coefficient matrix are added to prevent redundancy between samples/features from affecting the results and restrict the growth of the two matrices by their Frobenius norm. Firstly, the differentially expressed genes (DEGs) were obtained by differential analysis of the IRI-related data in the GEO database. Furthermore, the intersection of DEGs and pyroptosis-related genes (PRGs) was taken, and the intersection genes were input into the innovative algorithm proposed in this paper.

## 2. Methods

### 2.1. Nonnegative Matrix Factorization (NMF)

NMF algorithm is a classical dimension reduction method successfully applied in image recognition, speech recognition, biological data processing, and other fields. A complete matrix $X=\left[x_{1},x_{2}\ldots ,x_{n}\right]\in {\mathbb{R}}^{m\times n}$ is decomposed into two nonnegative matrices, including the base matrix $U=\left[u_{1},u_{2},\ldots u_{n}\right]\in {\mathbb{R}}^{m\times k}$ and the coefficient matrix $V=\left[v_{1},v_{2},\ldots v_{n}\right]\in {\mathbb{R}}^{k\times n}$, where m represents the number of features (samples), and n represents the number of samples (features). U and V store information about features and samples, respectively. In bioinformatics, omics data can be preprocessed to obtain the form of feature matrix X, which can be further applied to feature gene selection, disease subtype classification, and other tasks. The objective function of the NMF algorithm is shown below.

(1) $$O_{NMF}=\;∥X-UV{∥}_{F}^{2}\;s.t.\;U\geq 0,\;V\geq 0$$

Since this paper aims to analyze disease subtypes using (PRGs), m represents the number of genes, and n represents the number of samples.

### 2.2. GraphNet Regularizer

The GraphNet regularizer is a modified version of the elastic network regularization, which can integrate the connectivity information between samples or features. Specifically, if the connectivity between the i–th node and the j–th node is robust, GraphNet encourages this connectivity, making the two nodes more similar. The expression of the GraphNet regularizer is as follows.

(2) $$G\left(u\right)={\sum^{\;}}_{i,j}C_{u\left(i,j\right)}{\left(u_{i}-u_{j}\right)}^{2}\;G\left(v\right)={\sum^{\;}}_{i,j}C_{v\left(i,j\right)}{\left(v_{i}^{T}-v_{j}^{T}\right)}^{2}$$

Here, $u_{i}$ and $u_{j}$ represent the i–th and j–th sample points, respectively. $v_{i}$ and $v_{j}$ represent the i–th and j–th feature points, respectively. The Laplacian operator can rewrite the above equation.

(3) $$G\left(u\right)=U^{T}L_{u}U\;G\left(v\right)=VL_{v}V^{T}$$

Here, $L_{u}$ and $L_{v}$ represent the Laplaras matrix of X and Y, respectively. The Laplace matrix is defined as L=D−C. D is the degree matrix, and S stands for the connectivity matrix. Then, $L_{u}$ and $L_{v}$ can be expressed as the following equations, respectively.

(4) $$L_{u}=D_{u}-S_{u}\;L_{v}=D_{v}-S_{v}$$

### 2.3. Orthogonal Non-Negative Matrix Factorization Algorithm Based on Dual Laplace Regularization Constraint (DL-ONMF)

This paper adds the connectivity information between samples and features to the algorithm as prior knowledge. To reduce the influence of multicollinearity on the results, orthogonal constraints are applied to U and V, respectively. In addition, the Frobenius norm for U and V is also added based on the NMF algorithm to control the growth of U and V. The objective function of the DL-ONMF algorithm is as follows.

(5) $$\Gamma \left(U,V\right)=\min_{U,V}∥X-UV{∥}_{F}^{2}\;+\;\lambda_{1}∥V{∥}_{F}^{2}\;+\;\lambda_{2}∥U{∥}_{F}^{2}\;+\;\gamma_{1}\mathrm{Tr}\left(U^{T}L_{u}U\right)\;+\;\gamma_{2}\mathrm{Tr}\left(VL_{v}V^{T}\right)\;+\;\beta_{1}∥UU^{T}-I{∥}_{F}^{2}\;+\;\beta_{2}∥VV^{T}-I{∥}_{F}^{2}\;s.t.\;\;U,V\geq 0$$

where I represents the identity matrix, $\lambda_{1}$ and $\lambda_{2}$ control the growth of U and V, respectively. $\gamma_{1}$ and $\gamma_{2}$ control the strength of connectivity between features and samples, respectively. $\beta_{1}$ and $\beta_{2}$ control the power of the orthogonal constraints on U and V, respectively. The Lagrange multiplier method is used to optimize the objective function, as shown in Equation (6).

(6) $$L_{f}=\Gamma \left(U,V\right)+\mathrm{Tr}\left(\emptyset U^{T}\right)+\mathrm{Tr}\left(\varphi V^{T}\right)$$

Here, ∅ and φ are Lagrange multipliers. Next, $L_{f}$ takes the derivative with respect to U and V, respectively, to obtain Equation (7).

(7) $$\frac{\partial L_{f}}{\partial U}=-2XV+2UV^{T}V+2\lambda_{1}U+2\gamma_{1}L_{u}U+4\beta_{1}UU^{T}U-4\beta_{1}U+\emptyset \;\frac{\partial L_{f}}{\partial V}=-2X^{T}U+2VU^{T}U+2\lambda_{2}V+2\gamma_{2}L_{v}V+4\beta_{2}VV^{T}V-4\beta_{2}V+\varphi$$

Let the partial derivative be zero, and the iteration rules for U and V can be obtained.

(8) $$\begin{array}{c} u_{ik}\leftarrow u_{ik}\frac{{\left(XU\;+\;\gamma_{1}S_{u}U\;+\;2\beta_{1}U\right)}_{ik}}{{\left(UV^{T}V\;+\;\lambda_{1}U\;+\;\gamma_{1}D_{u}U\;+\;2\beta_{1}UU^{T}U\right)}_{ik}}\\ v_{ik}\leftarrow v_{ik}\frac{{\left(XU\;+\;\gamma_{1}S_{v}U\;+\;2\beta_{2}V\right)}_{ik}}{{\left(VU^{T}U\;+\;\lambda_{2}V\;+\;\gamma_{1}D_{v}V\;+\;2\beta_{2}VV^{T}V\right)}_{ik}} \end{array}$$

### 2.4. Definition of Reconstruction Performance of the NMF Algorithm

Since the NMF-based algorithms decompose the expression matrix into two (or more) non-negative matrices in the form of multiplication, the correlation between the original expression matrix and the multiple matrices after dimensionality reduction can measure the performance of the algorithm for data reconstruction. In this paper, the reconstruction performance of the algorithm is calculated using the Pearson correlation coefficient (PCC), which is PCC(X,UV). In addition, the relative error is introduced to measure the performance of the algorithm.

(9) $$relative\_error=\;∥X-UV{∥}_{F}^{2}$$

### 2.5. Silhouette Coefficient

The silhouette coefficient is a way to evaluate the clustering effect. The contour coefficients were calculated using the following formula.

(10) $$s\left(i\right)=\frac{b\left(i\right)-a\left(i\right)}{max\left\{a\left(i\right),b\left(i\right)\right\}}$$

Here, a(i) is the average distance between sample i and other samples in the same cluster. The smaller a(i) is, the more sample i should be clustered to that cluster. Let a(i) be called the intra-cluster dissimilarity of sample i. b(i) is the average distance between sample i and all samples of some other cluster. The contour coefficients take values between −1 and 1. The best value is 1, and the worst value is −1. Values close to 0 indicate overlapping clusters. Negative values usually indicate that the sample are assigned to the wrong cluster because different clusters are more similar.

### 2.6. PRGs Expression before and after Renal Ischemia-Reperfusion

The “limma” package was used to explore the differential expression of 110 PRGs in GSE43974, GSE126805, and their combined datasets. p-value < 0.05 and log FC > 0 were used as the threshold for screening differentially expressed PRGs (DEPRGs). The differential results associated with PRGs in the pooled dataset were then visualized in heat maps and volcano maps. The differential results associated with PRGs in GSE43974 and GSE126805 are visualized in boxplots. Protein–protein interaction (PPI) networks were constructed to assess gene interactions among DEPRGs and visualized in Cytoscape. To determine the correlation between pairs of DEPRGs, Pearson’s correlation coefficients were calculated for DEPRGs in samples after IRI from the pooled dataset and visualized using “complot” in R.

### 2.7. Enrichment Analysis and Immune Analysis of Different Clusters

Gene ontology (GO) enrichment analysis (including biological process (BP), cellular component (CC), and molecular function (MF)) and Kyoto Encyclopedia of Genes and Genomes (KEGG) enrichment analysis were performed to explore and compare the enrichment functions of differentially expressed genes among subtypes. The R package “cluster profile” was used to perform KEGG and GO analyses. Single-Sample Gene Set Enrichment Analysis (ssGSEA) was used to calculate the differences in immune pathways and cells in different renal ischemia-reperfusion injury subtypes. In addition, we evaluated the expression differences of PRGs, immune checkpoint loci, and HLA-related genes among different subtypes, which were visualized using box plots.

### 2.8. Construction and Validation of Renal Transplantation Related Diagnostic Model

Two machine learning algorithms, least absolute shrinkage and selection operator (LASSO), and support vector machine recursive feature elimination (SVM-RFE), were used to screen feature genes. The “glmnet” package was used to perform LASSO analysis, and ten-fold cross-validation was used to avoid overfitting. SVM-RFE was used to rank PRGs related to renal transplant progression. SVM-RFE was used for feature selection by tenfold cross-validation. “pROC” was used to draw ROC curves to evaluate the diagnostic performance of the diagnostic model and diagnosis-related PRGs.

### 2.9. Construction of Nomogram

Nomogram construction was precious for the diagnosis of clinical renal transplantation outcomes. Based on diagnosis-related PRGs, the “rms” R package was applied to construct the nomogram. Calibration curves were used to assess the accuracy of the nomogram. Decision curve analysis was used to evaluate the clinical utility of the nomogram. The clinical impact curve was drawn to predict high-risk probability stratification for a population of 1000.

## 3. Results

### 3.1. Data Preprocess

Two kidney-transplantation-related datasets, GSE21374 and GSE36059, were downloaded from GEO database. In GSE21374, samples of successful and failed kidney transplants were included. GSE36059 included samples of successful and failed kidney transplants. GSE21374 was used as the training dataset of the diagnostic model, and GSE36059 was used as the test dataset to verify the prediction accuracy of the diagnostic model.

The “SVA” package was used to eliminate batch effects of GSE43974 and GSE126805. Next, GSE43974 and GSE126805 were integrated, resulting in 205 and 246 samples after renal I/R. In addition, a total of 110 pyroptosis-related genes (PRGs) were collected from previous papers [12] (Supplementary Material Figure S1). Finally, the expression data of 110 PRGs were extracted from GSE43974, GSE126805, and their combined datasets, respectively.

### 3.2. Identification of DEPRGs in Renal Ischemia-Reperfusion Injury

**Figure 1:**
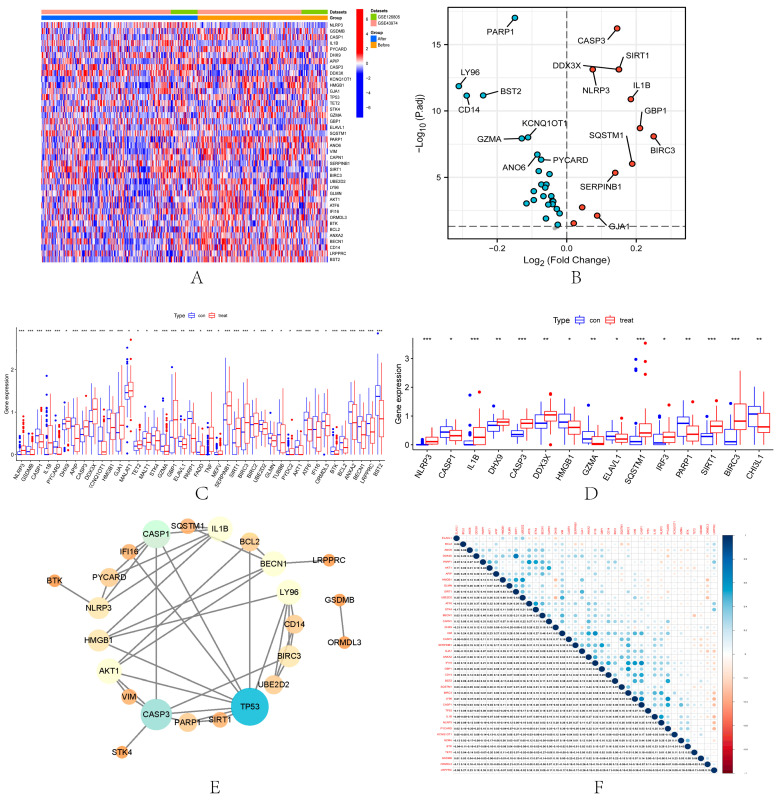
Identification of DEPRGs in IRI. Heatmap (**A**) and volcano map (**B**) of 40 DEPRGs in the combined dataset. Boxplots show the difference in DEPRGs expression before and after renal ischemia-reperfusion in GSE43974 (**C**) and GSE126805 (**D**). * *p* < 0.05, ** *p* < 0.01, the *** *p* < 0.001. (**E**) PPI networks of 40 DEPRGs in the combined dataset. (**F**) Correlation among 40 DEPRGs in the combined dataset. Positive correlations are shown in blue and negative correlations are in red. The color depth reflects the strength of the correlation.

A total of 74 PRGs were extracted from the combined dataset. Among the 74 PRGs, 40 DEGs were identified from the combined dataset (Figure 1A,B). Among them, the expression of 28 DEPRGs was down-regulated in IRI samples compared with samples before renal ischemia-reperfusion, including LY96, CD14, BST2, PARP1, GZMA, VIM, KCNQ1OT1, CAPN1, ANXA2, ANO6, CASP1, PICARD, APIP, APIP, GZMA. HMGB1, LRPPRC, IFI16, ATF6, BECN1, AKT1, UBE2D2, ELAVL1, STK4, BCL2, ORMDL3, BTK, TP53, GLMN and GSDMB. Twelve DEPRGs were up-regulated in IRI, including TET2, DHX9, NLRP3, GJA1, SERPINB1, CASP3, DDX3X, SIRT1, IL1B, SQSTM1, GBP1, and BIRC3. In addition, 41 and 15 DEGs were identified in the GSE43974 dataset (Figure 1C) and GSE126805 dataset (Figure 1D), respectively. The PPI network revealed the interactions among 40 IRI-related PRGs (Figure 1E). TP53 had the highest central position in the PPI interaction network. Correlation analysis showed the correlation of 40 PRGs in IRI (Figure 1F). SIRT1 had the highest correlation with DDX3X (cor = 0.72).

### 3.3. Selection of Hyperparameters for DL-ONMF Algorithm

**Figure 2:**
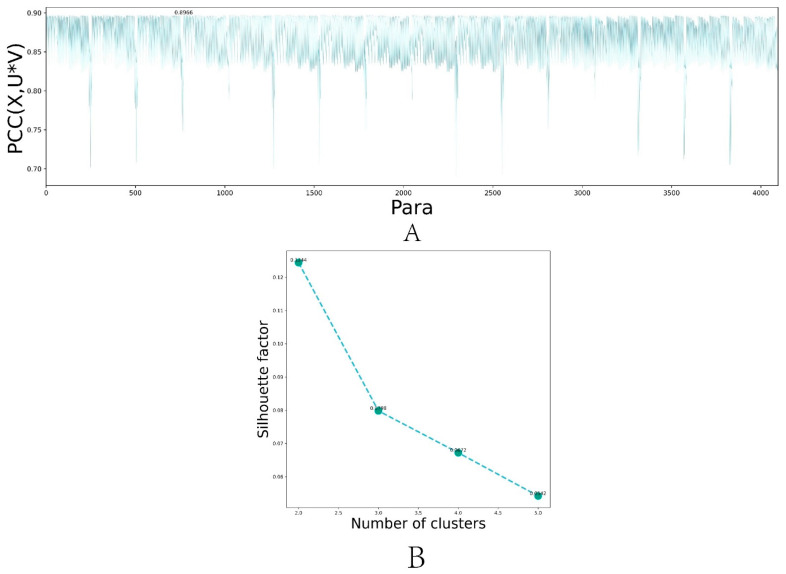
Variation trend of hyperparameter selection and function values of different constraint terms with the increasing number of iterations for DL-ONMF algorithm. (**A**) Line plot of the reconstruction performance of the algorithm under different parameter combinations. (**B**) Line plot of the reconstruction performance of the algorithm for different values of k.

The DL-ONMF algorithm proposed in this paper is assumed to involve six parameters $\lambda_{1}$, $\lambda_{2}$, $\gamma_{1}$, $\gamma_{2}$, $\beta_{1}$, and $\beta_{2}$. A parameter set [0.001 0.01 0.1 1] was set. The six hyperparameters were selected among the four specified parameters using a grid search method. We showed the dimension reduction k for the selection process in Figure 2A. The dimension reduction performance of the algorithm was reduced due to the excessive setting. Therefore, the value of k was set between 1 and 5, and the reconstruction performance of the algorithm with different values of k was calculated. Finally, k was set to 5 in this paper. Next, we fixed the best k value and selected the other hyperparameters. Figure 2B showed the reconstruction performance of the algorithm under different parameter combinations. Among them, the PCC corresponding to the 769th group of parameters was the largest, reaching 0.8966. We select the best parameters combination for $\lambda_{1}=0.001$, $\lambda_{2}=1$, $\gamma_{1}=0.001$, $\gamma_{2}=0.001$, $\beta_{1}=0.001$ and $\beta_{2}=0.001$. After 100 iterations of the algorithm, the function values of all constraint terms tended to be stable.

### 3.4. Algorithm Clustering Results

**Figure 3:**
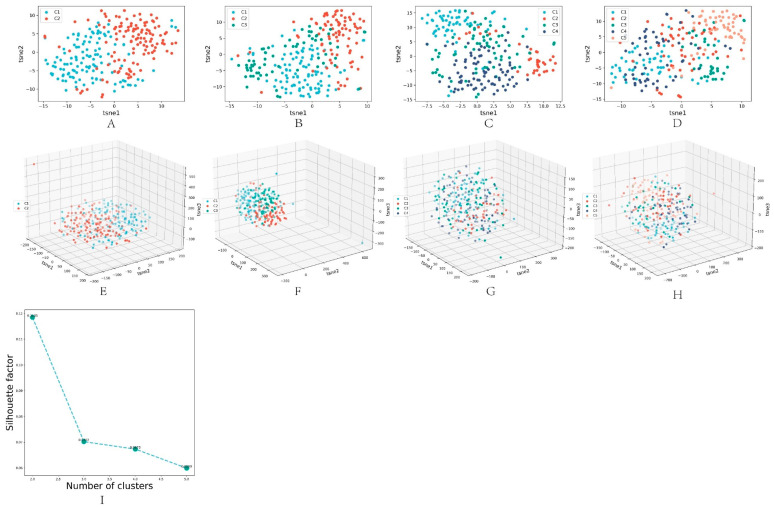
Results obtained under the best parameters for different cluster numbers. (**A**–**D**) are 2D visualized scatter plots of other cluster numbers (2 to 5) obtained using the t-sne dimensionality reduction algorithm. (**E**–**H**) are 3D visualization scatter plots of different cluster numbers (2 to 5) obtained using the t-sne dimensionality reduction algorithm. (**I**) is the line plot of the contour coefficient change as the number of clusters increases.

This paper used the spectral clustering [13] method to obtain the final clustering results for the dimension reduction results obtained by the DL-ONMF algorithm. Specifically, this paper set the number of clusters from 2 to 5, and the contour coefficients were obtained under different cluster numbers. Figure 3A–D illustrated the 2D visualized scatter plots obtained using the t-sne dimensionality reduction algorithm for other cluster numbers (2 to 5). Figure 3E–H showed the 3D visualization scatter plots for different cluster numbers (2 to 5) obtained using the t-sne dimensionality reduction algorithm. The other colored points represented different classes of samples. In addition, this paper showed the contour coefficients for different cluster numbers in Figure 3I. The highest contour coefficients were obtained when clustering into two classes. Therefore, we set the number of clusters to two.

### 3.5. Comparison of Algorithm Performance

In order to evaluate the performance of the proposed algorithm, two metrics, reconstruction performance and reconstruction error, are introduced in Section 2.4. Table 1 presents the performance comparison results of the two algorithms.

In addition, in order to evaluate the clustering performance of NMF, K-means and DL-ONMF, we counted the visualization and contour coefficient results of t-sne and PCA dimensionality reduction, respectively, when the clustering of the three algorithms was in 2–5 classes. Among them, the visualization results of t-sne dimensionality reduction and contour coefficient changes of DL-ONMF algorithm are shown in Figure 3. Other cases are shown in the supplementary material. As can be seen in Figure 3 and Figures S1–S3 in the supplementary material, the proposed algorithm obtains the highest contour coefficients in both classes of cases. In addition, it can be seen from the dimensionality reduction visualization that the proposed algorithm has a clearer clustering effect in the two types of cases.

### 3.6. Subtype Analysis

**Figure 4:**
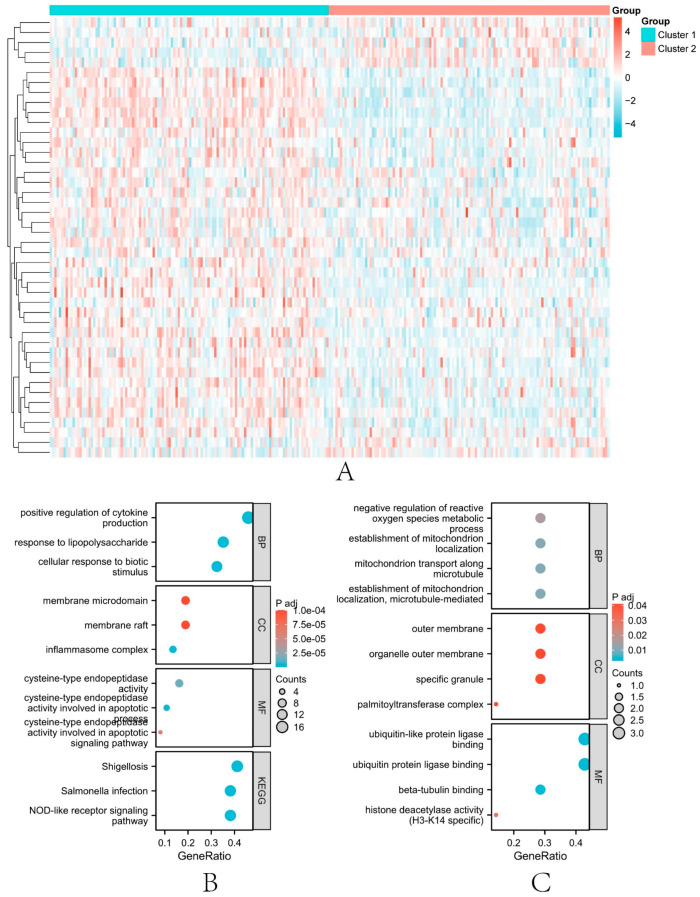
Results of differential and enrichment analysis of genes in the two subtype groups. (**A**) 44 differentially expressed genes (p < 0.05) in the two subtype groups. (**B**) the bubble plot of the GO pathway and KEGG pathway in which up-regulated genes are involved. (**C**) the bubble plot of the GO pathway and KEGG pathway in which down-regulated genes are involved.

First, we performed a difference analysis using the limma algorithm on the two obtained subtype groups, with a p value set at 0.05 (*t*.test). Finally, 44 differentially expressed genes were obtained. A heat map of the differential expression of these genes was generated (Figure 4A). Among these genes, 37 genes were up-regulated and 7 genes were down-regulated. GO and KEGG enrichment analysis were performed for up-regulated genes and down-regulated genes, respectively, and bubble plots of enrichment results were drawn (Figure 4B,C).

### 3.7. Different Immune Characteristics among IRI Subtypes

**Figure 5:**
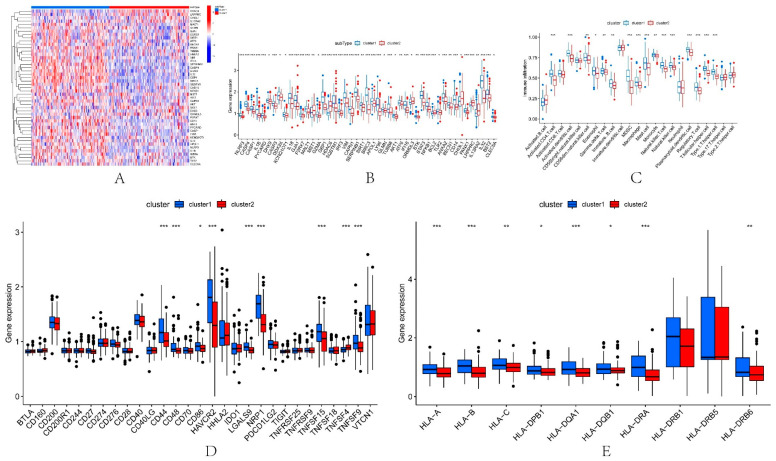
Immune features in the two pyroptosis-related clusters. Heatmap (**A**) and boxplot (**B**) of 47 DEPRGs in the typing dataset. (**C**) The difference in the abundance of immune cells between cluster1 and cluster2. (**D**) Expression differences between cluster1 and cluster2. (**E**) Expression differences of HLA-related genes between cluster1 and cluster2. * *p* < 0.05, ** *p* < 0.01, *** *p* < 0.001.

Firstly, the expression of 74 PRGs in the two pyroptosis-related subtypes was elucidated, and a total of 47 PRGs were differentially expressed (wilcox.test) between the two subtypes (Figure 5A,B). Previous studies have shown that IRI involves innate and adaptive immune responses [14]. Therefore, in this paper, we used ssGSEA to explore the immunological characteristics among IRI subtypes. The results of immunoassays showed that the abundance of a variety of immune cells was different between subtypes. Such as Activated.CD4.T.cell, Activated.dendritic.cell, CD56dim.natural.killer.cell and Gamma.delta.T.cell (Figure 5C). We found that the abundance of immune cells in IRI patients was generally higher in cluster1 than in cluster2. In addition, 48 immunological examination sites and 25 HLA-related genes were also explored in the two subtypes. The results showed that a total of 9 immune examination sites (Figure 5D) and 8 HLA-related genes (Figure 5E) were differentially expressed between cluster1 and cluster2, and their expression in cluster1IRI patients was also higher than that in cluster2. These results indicate that this paper’s immune microenvironment of the two pyroptosis-related IRI subtypes identified by the DL-ONMF algorithm is significantly different and may be an essential factor in kidney transplantation. Therefore, it is necessary to further explore the effect of the two subtypes of DEPRGs on the success and failure of kidney transplantation.

### 3.8. Construction of Renal Transplantation Related Diagnostic Model

**Figure 6:**
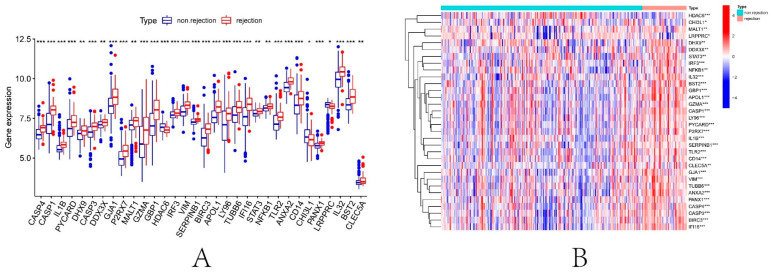
Identification of DEPRGs in kidney transplantation. (**A**) Boxplot of 33 DEPRGs expression. (**B**) Expression heatmap of 33 DEPRGs. * *p* < 0.05, ** *p* < 0.01, *** *p* < 0.001.

To explore the significance of DEPRGs in the process of renal transplantation, the expression of 47 DEPRGs in successful and failed renal transplantation patients was elucidated. In Figure 6A,B, 33 of 47 PRGs were differentially expressed between the successful and failed renal transplant recipients (*p* < 0.05). The expression profiles of these 33 DEPRGs were left as input for the following analysis.

**Figure 7:**
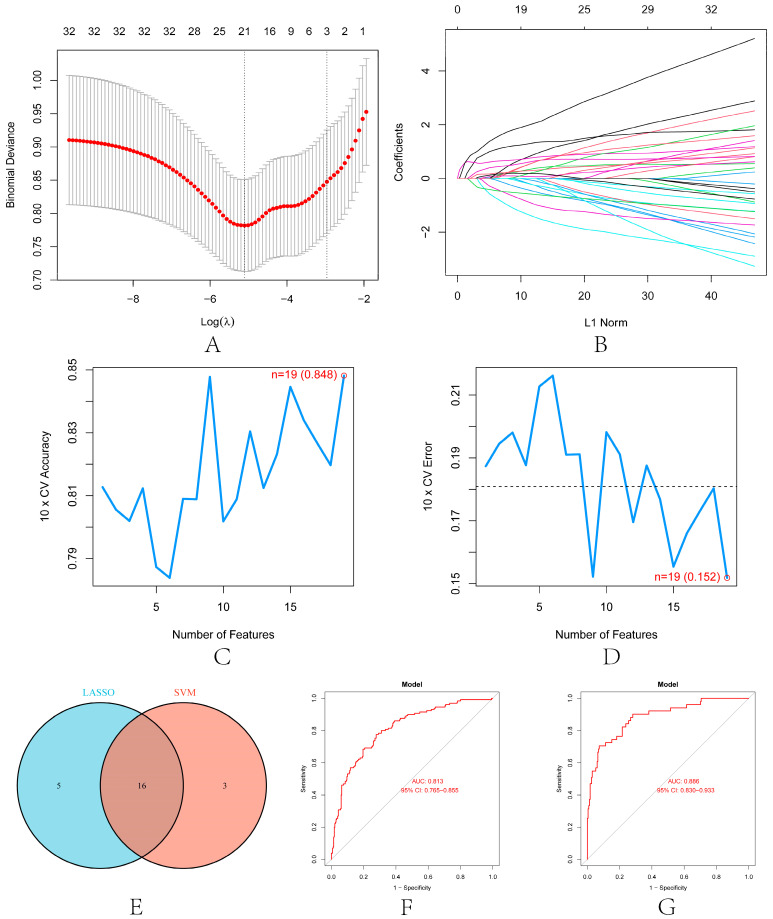
Machine learning in screening candidate diagnostic biomarkers for renal transplantation. (**A**) Plot of partial likelihood deviance. (**B**) Plot of LASSO coefficient profiles. Accuracy (**C**) and error (**D**) of 10-fold cross-validation (CV) in SVM-RFE algorithms, respectively. (**E**) Venn diagram showing the characteristic genes shared by LASSO and SVM-RFE algorithms. ROC curve evaluates the diagnostic performance of characteristic genes in GSE21374 (**F**) and GSE36059 (**G**).

**Figure 8:**
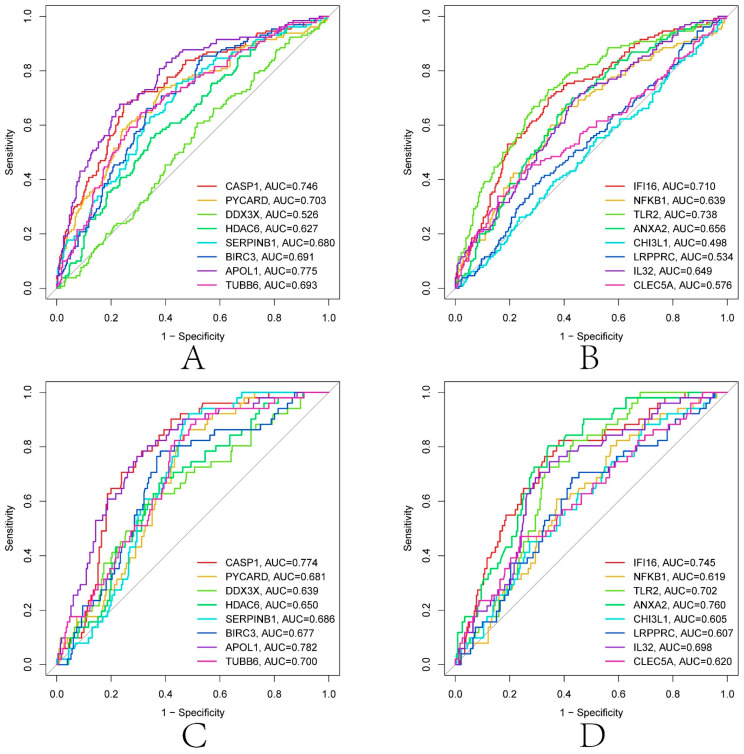
Diagnostic performance of 16 diagnosis related genes in GSE21374 and GSE36059. (**A**,**B**) GSE21374. (**C**,**D**) GSE36059.

LASSO and SVM-RFE were used to screen further the diagnostic features related to renal transplantation. The partial likelihood deviations and coefficients of LASSO versus logλ show (Figure 7A,B) that 21 diagnosis-relevant PRGs (CASP1, IL1B, PYCARD, DDX3X, GJA1, GBP1, HDAC6, SERPINB1, BIRC3, APOL1, *p* < 0.05) were identified. TUBB6, IFI16, STAT3, NFKB1, TLR2, ANXA2, CHI3L1, LRPPRC, IL32, BST2, and CLEC5A). According to the importance of SVM-RFE calculation and ten-fold cross-validation results (Figure 7C,D), 19 kidney transplant diagnosis-related PRGs (ANXA2, CASP1, TLR2, PYCARD, DDX3X, IL32, APOL1, SERPINB1, CLEC5A, NFKB1, and PRGS (ANXA2, CASP1, TLR2, PYCARD, DDX3X, IL32) were identified. LRPPRC, CHI3L1, TUBB6, HDAC6, GZMA, CD14, BIRC3, IFI16, and DHX9). Then, we intercrossed the diagnostic genes screened by the two algorithms, and a total of 16 renal transplantation diagnosis-related PRGs (CASP1, PYCARD, DDX3X, HDAC6, SERPINB1, BIRC3, APOL1, TUBB6, IFI16, NFKB1, TLR2, ANXA2, PRGS) were obtained. CHI3L1, LRPPRC, IL32, and CLEC5A) (Figure 7E). To evaluate the predictive accuracy of the diagnostic model, ROC curves were plotted for the GSE21374 and GSE36059 datasets. The AUC of GSE21374 and GSE36059 was 0.886 and 0.813, respectively (Figure 7F,G). Finally, we plotted separate ROC curves for the 16 diagnostically relevant PRGs. In Figure 8A–D, 16 genes also showed better diagnostic performance in GSE21374 and GSE36059 alone. Specifically, CASP1 had the best diagnostic performance, with an AUC of 0.774 in the ROC curve of the GSE21374 dataset.

### 3.9. Construction of Nomogram

**Figure 9:**
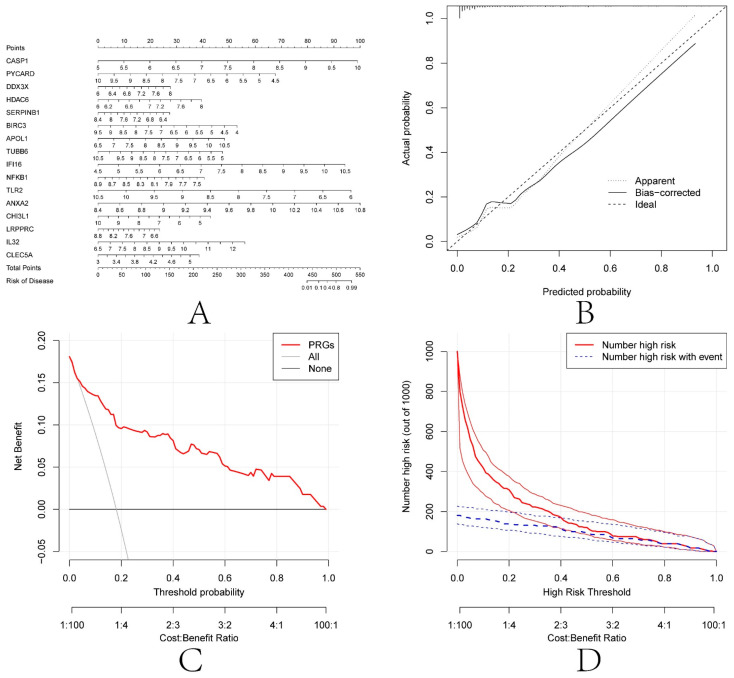
Construction of the nomogram model. (**A**) Construction of the nomogram model based on the selected PRGs (CASP1, PYCARD, DDX3X, HDAC6, SERPINB1, BIRC3, APOL1, TUBB6, IFI16, NFKB1, TLR2, ANXA2, CHI3L1, LRPPRC, IL32 and CLEC5A). (**B**) Calibration curve illustrating the diagnostic ability of the nomogram model. (**C**) Nomogram models have higher clinical utility than individual PRGs, according to DCA. (**D**) The clinical impact curve demonstrates a high level of diagnostic ability of the nomogram model.

To further explore these 16 diagnosis-related PRGs (CASP1, PYCARD, DDX3X, HDAC6, SERPINB1, BIRC3, APOL1, TUBB6, IFI16, NFKB1, TLR2, ANXA2, CHI3L1, LRPPRC, IL32, and CLEC5A), a nomogram model was constructed based on these 16 genes (Figure 9A). The calibration curve showed that the nomogram model based on the 16 diagnosis-related PRGs was in good agreement with the ideal model (Figure 9B). DCA analysis showed that although both the nomogram model and individual diagnosis-related PRGs generated net benefits, the net use of the nomogram model was significantly greater than that of individual diagnosis-related PRGs, indicating that the nomogram model may be more clinically useful than individual diagnosis-related PRGs (Figure 9C). Analysis of the clinical impact curves showed that the nomogram model had relatively high diagnostic power (Figure 9D).

## 4. Discussion

IRI often occurs in the process of kidney transplantation and affects the renal function and prognosis of patients. This paper proposes a DL-ONMF algorithm to classify patients with IRI and confirm two IRI subtypes. The two IRI subtypes have significant differences in immune characteristics. Specifically, we first extracted differentially expressed genes and the intersection genes of 110 PRGs. The PPI network was used to reveal the interactions among 40 IRI-related PRGs. We obtained DEGs between the two isoforms using the DL-ONMF algorithm under the best parameter combination. This study found that most of the pathways enriched by differential genes in the two groups were closely related to reperfusion injury. We found that most of the significant pathways involved in up-regulated genes were closely related to the treatment of renal ischemia. For example, G protein α12 is a negative regulator of adipocyte mediated by kidney injury molecule-1, which can inhibit the activation of reactive oxygen species during renal ischemia-reperfusion injury [15]. G-protein-coupled receptor 35 agonists can realize mitochondrial remodeling and renal ischemia protection [16]. On the contrary, most of the significant pathways involved in down-regulated genes were closely related to the development of renal ischemia/renal disease. For example, In the treatment experiments of ischemia-reperfusion injury in transgenic and wild-type mice, transgenic mice showed rapid and enhanced renal injury. Transgenic mice with low Igf-1Ea expression can significantly up-regulate pro-inflammatory cytokines such as TNF-α and Ccl2 [17]. The inflammasome is an attractive potential therapeutic target in a variety of renal diseases [18].

The immune analysis between IRI subtypes by the ssGSEA algorithm showed that the abundance of immune cells and the abundance of 9 immune check sites were different between the two subtypes. To explore the significance of DEPRGs in the process of renal transplantation among PRGS-related IRI subtypes, 47 DEPRGs were analyzed in the successful and failed renal transplantation. The obtained DEGs were put into the LASSO and SVM-RFE algorithms, respectively. The intersection of diagnostic genes screened by the two algorithms was taken, and 16 PRGs related to kidney transplantation diagnosis were obtained. Vasantha et al. demonstrated that CASP1 is a driver of hepatocyte injury in fatty livers undergoing IRI and that its inhibition leads to liver protection [19]. Yuan et al. confirmed the potential role of HDAC6 in the retinal IRI model through biological experiments [20]. Toll-like receptor (TLR) plays a central role in recognizing pathogens and damage-associated molecular patterns in innate immunity. F Arslan et al. summarized the beneficial effects of therapeutic inhibition of TLR2 on IRI injury in a mouse model of myocardial infarction [21]. Dmitry et al. identified candidate genes, including ANXA2, by performing an in situ renal IRI meta-analysis of 150 microarray samples [22]. Deng et al. found that upregulation of miR-381-5p enhanced the effect of dexmedetomidine preconditioning in preventing myocardial ischemic IRI in a mouse model by inhibiting CHI3L1 [23]. The study by Zhou et al. confirmed that LRPPRC as a transcription factor might be an essential target for protection against IRI injury mediated by rhizoma alkaloids in Coptis rhizoma [24]. In addition, PYCARD, NFKB1, and IL32 may also play a role in related diseases induced by IRI [22,25,26,27].

Finally, a nomogram model was constructed for the 16 genes, and the clinical implications of the 16 diagnosis-related PRGs were explored. The results showed that the nomogram model had relatively high diagnostic power.

## 5. Conclusions

As a severe complication of transplantation, IRI has different subtypes of prognosis. In this paper, we proposed a novel DL-ONMF clustering method to reduce the influence of redundant features while entirely using the prior information contained in PRGs. We performed an exhaustive biogenic analysis of the two subtypes obtained by the DL-ONMF algorithm. The immunoassay results showed that the two PRGS-related subtypes obtained by the DL-ONMF algorithm had different immune characteristics. Specifically, patients with IRI in type 1 had a generally higher immune response than those with IRI in type 2. This suggests that inter-subtyping differences may be contributing factors to the success or failure of renal transplantation. Based on the inter-subtype DEPRGs, we used LASSO and SVM-RFE algorithms to construct a diagnostic model related to renal transplantation. The diagnostic model could reasonably predict the outcome of kidney transplant patients (AUC = 0.886). In the subsequent study, we consider using the patient’s clinical information as a priori information to induce the algorithm to cluster IRI subtypes closely related to clinical indicators and provide new insights into the precise treatment of IRI.

## Figures and Tables

**Table 1:** Results of algorithm performance comparison.

Algorithm	Reconstruction Performance	Reconstruction Error
NMF	0.8955	8.2945
DL-ONMF	0.8964	8.2773

## Data Availability

The data presented in this study are openly available in GEO dataset (https://www.ncbi.nlm.nih.gov/geo/).

## Supplementary Materials

The following supporting information can be downloaded at: https://www.mdpi.com/article/10.3390/biom13020275/s1, Figure S1: The results obtained by the NMF algorithm under the best parameters for different cluster numbers. (A–D) are 2D visualized scatter plots of other cluster numbers (2 to 5) obtained using the t-sne dimensionality reduction algorithm. (E–H) are 3D visualization scatter plots of different cluster numbers (2 to 5) obtained using the t-sne dimensionality reduction algorithm; Figure S2: The results obtained by the K-means algorithm under the best parameters for different cluster numbers. (A–D) are 2D visualized scatter plots of other cluster numbers (2 to 5) obtained using the t-sne dimensionality reduction algorithm. (E–H) are 3D visualization scatter plots of different cluster numbers (2 to 5) obtained using the t-sne dimensionality reduction algorithm. (I) is the line plot of the contour coefficient change as the number of clusters increases; Figure S3: The results obtained by the NMF algorithm under the best parameters for different cluster numbers. (A–D) are 2D visualized scatter plots of other cluster numbers (2 to 5) obtained using the PCA dimensionality reduction algorithm. (E–H) are 3D visualization scatter plots of different cluster numbers (2 to 5) obtained using the PCA dimensionality reduction algorithm. (I) is the line plot of the contour coefficient change as the number of clusters increases; Figure S4: The results obtained by the K-means algorithm under the best parameters for different cluster numbers. (A–D) are 2D visualized scatter plots of other cluster numbers (2 to 5) obtained using the PCA dimensionality reduction algorithm. (E–H) are 3D visualization scatter plots of different cluster numbers (2 to 5) obtained using the PCA dimensionality reduction algorithm. (I) is the line plot of the contour coefficient change as the number of clusters increases; Figure S5: Comparison of the contour coefficients of the three algorithms. (A–C) show the line plots of the contour coefficients of NMF, K-means and DL-ONMF as the number of clusters, respectively.
