# Supplementary material for: Identification of the Subtypes of Renal Ischemia-Reperfusion Injury Based on Pyroptosis-Related Genes
Source: Biomolecules. 2023 Feb 1;13(2):275. doi: 10.3390/biom13020275 (PMC9952921; doi:10.3390/biom13020275)
Supplement: Supplementary file 1 [file biomolecules-13-00275-s001.zip › biomolecules-2017566-supplementary.pdf]

Supplementary Material

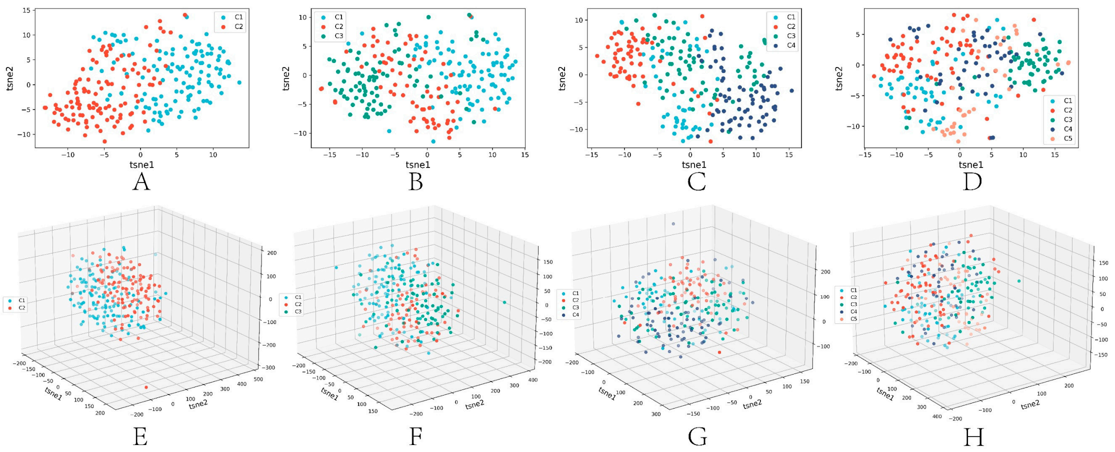

Fig. S1 The results obtained by the NMF algorithm under the best parameters for different cluster numbers. (A)-(D) are 2D visualized scatter plots of other cluster numbers (2 to 5) obtained using the t-sne dimensionality reduction algorithm. (E)-(H) are 3D visualization scatter plots of different cluster numbers (2 to 5) obtained using the t-sne dimensionality reduction algorithm.

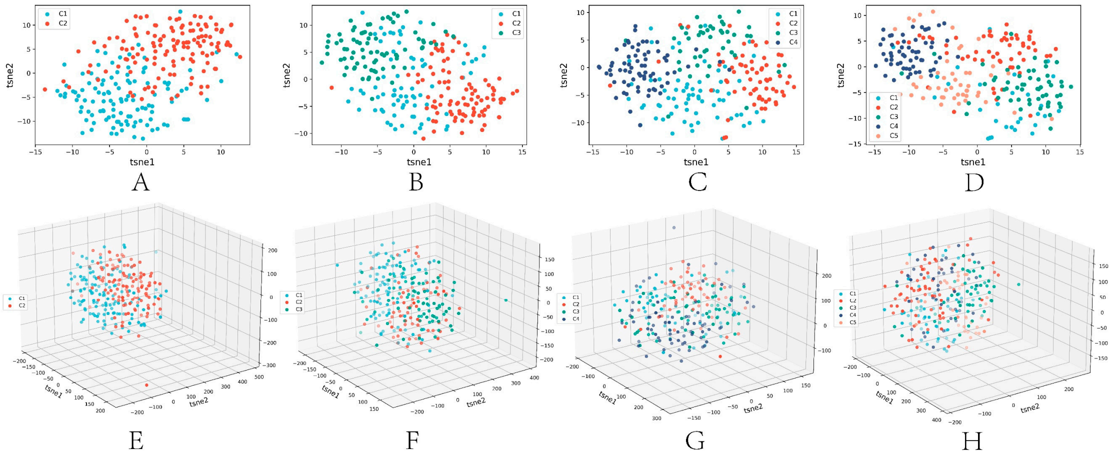

Fig. S2 The results obtained by the K-means algorithm under the best parameters for different cluster numbers. (A)-(D) are 2D visualized scatter plots of other cluster numbers (2 to 5) obtained using the t-sne dimensionality reduction algorithm. (E)-(H) are 3D visualization scatter plots of different cluster numbers (2 to 5) obtained using the t-sne dimensionality reduction algorithm. (I) is the line plot of the contour coefficient change as the number of clusters increases.

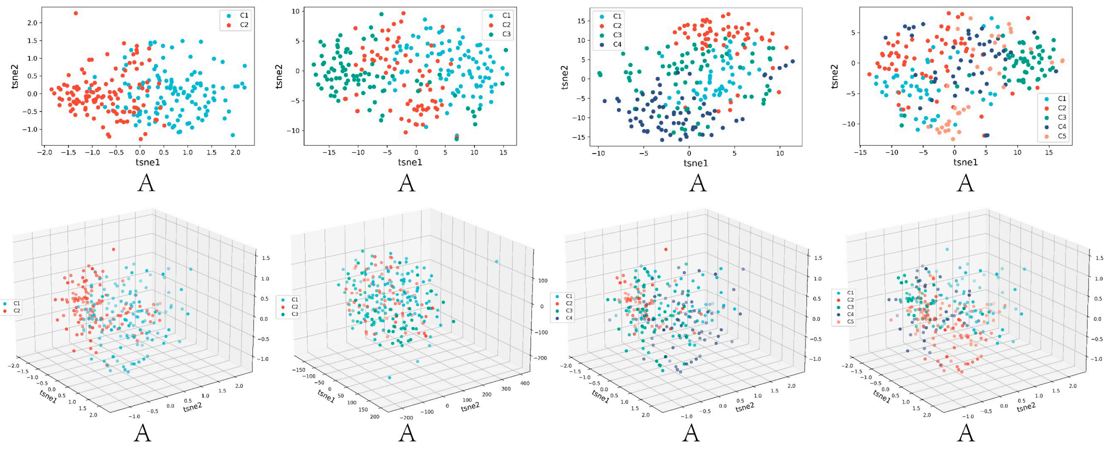

Fig. S3 The results obtained by the NMF algorithm under the best parameters for different cluster numbers. (A)-(D) are 2D visualized scatter plots of other cluster numbers (2 to 5) obtained using the PCA dimensionality reduction algorithm. (E)-(H) are 3D visualization scatter plots of different cluster numbers (2 to 5) obtained using the PCA dimensionality reduction algorithm. (I) is the line plot of the contour coefficient change as the number of clusters increases.

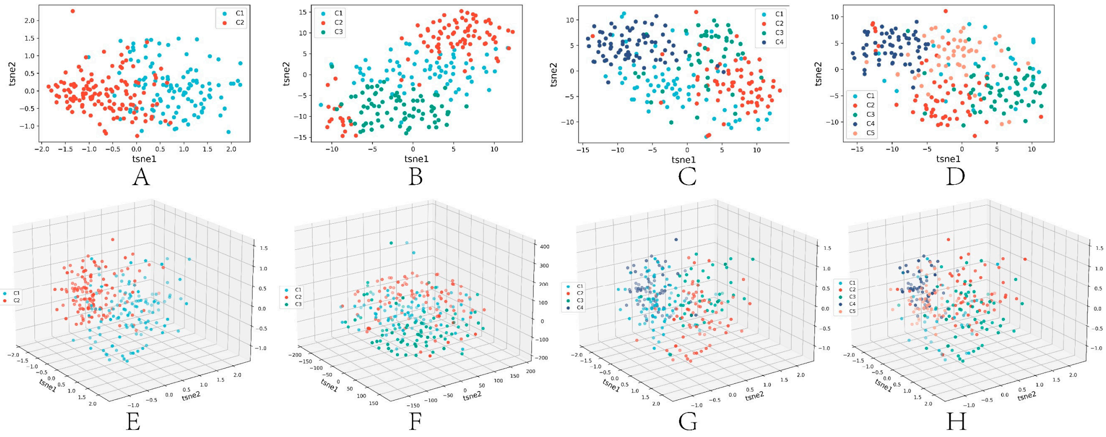

Fig. S4 The results obtained by the K-means algorithm under the best parameters for different cluster numbers. (A)-(D) are 2D visualized scatter plots of other cluster numbers (2 to 5) obtained using the PCA dimensionality reduction algorithm. (E)-(H) are 3D visualization scatter plots of different cluster numbers (2 to 5) obtained using the PCA dimensionality reduction algorithm. (I) is the line plot of the contour coefficient change as the number of clusters increases.

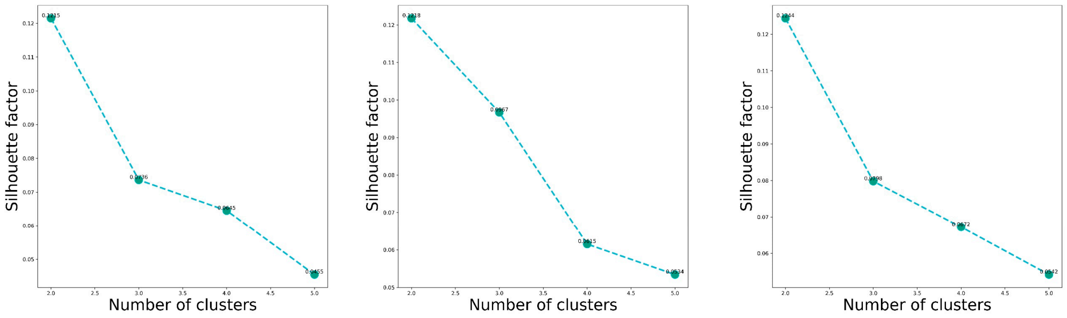

A B C

Fig. S5 Comparison of the contour coefficients of the three algorithms. A-C show the line plots of the contour coefficients of NMF, K-means and DL-ONMF as the number of clusters, respectively.
